# Editorial: Endocrine Disrupters and Metabolism

**DOI:** 10.3389/fendo.2019.00859

**Published:** 2019-12-10

**Authors:** Yann Gibert, Robert M. Sargis, Angel Nadal

**Affiliations:** ^1^Department of Cell and Molecular Biology, University of Mississippi Medical Center, Jackson, MS, United States; ^2^Division of Endocrinology, Diabetes, and Metabolism, Department of Medicine, University of Illinois at Chicago, Chicago, IL, United States; ^3^Instituto de Investigación, Desarrollo e Innovación en Biotecnologia Sanitaria de Elche, IDiBE and CIBERDEM, Universitas Miguel Hernández, Elche, Spain

**Keywords:** endocrine disrupters, metabolism, metabolic diseases, lipids, diabetes, pancreas, adipose tissue

Endocrine-disrupting chemicals (EDCs) are compounds of natural or human-made origin that are capable of interfering with the endocrine system of an organism (1, 2). EDCs can mimic or inhibit naturally occurring hormones by binding classical nuclear hormone receptors or by disrupting other pathways regulating hormone synthesis or action, thereby disrupting the normal physiology and homeostasic processes of the organism (3, 4). In recent years, increasing clinical, experimental, developmental and physiological data indicate that EDCs disrupt cellular and whole-body metabolism (5). Indeed, recent research has identified exposures to these metabolism-disrupting chemicals (MDCs) as playing a causative role in a wide spectrum of metabolic disorders in humans, including obesity, diabetes, dyslipidemia, nonalcoholic fatty liver disease (NAFLD), and cardiovascular dysfunction (5, 6).

The scope of the present Research Topic, including, 6 mini-review articles, 9 review articles, and 6 original papers, is to provide new insights into the field of endocrine disrupter biology and their impact on metabolic function and disease risk. This assemblage of work from experts in the field make clear the burgeoning data implicating MDCs in metabolic disease pathogenesis and provide a clarion call for action to address this underappreciated driver of metabolic disease risk.

## Original Research Articles

Balise et al. present a research paper in which they investigated the endocrine-disrupting effects of chemicals used in unconventional oil and gas (UOG) operations. By exposing pregnant mice to these MDCs, they noted an increase in energy expenditure. They investigated whether aging and metabolic stress exacerbate the impact of these MDCs on energy expenditure and observed an increase of activity and non-resting energy expenditure in mice exposed to MDCs using in UOG extraction.

Bastos-Sales et al. analyze the effects of perinatal administration of diethylhexyl phthalate (DEHP) on the F1 offspring during a one-year follow-up. The authors used a wide range of doses to find sex-dependent changes in lipid metabolism, including increases of free fatty acids and high-density lipoprotein cholesterol (HDL-C) without changes in weight, fat mass or glucose homeostasis-related parameters. Alterations of behavior and immune function were observed at the highest concentrations tested. These results support the dyslipidemic actions of developmental exposures to DEHP.

In original research, Fleisch et al. explore the association between prenatal exposure to air pollutants and body mass index (BMI) trajectories from birth to mid-childhood. Using Project Viva data from the Boston area, the authors determined that prenatal air pollution exposure did not predict early life weight gain in this cohort of moderately exposed individuals.

Cabaton et al. used metabolomics to investigate the modulation of human hepatic cell (HepG2) by the MDC bisphenol A (BPA) and the natural hormone 17β-estradiol (E2). This original research article demonstrates that the combined use of metabolomics with network reconstruction is a powerful technique when applied to *in vitro* studies to compare commonalities and differences in the mode of action of EDCs. Here they have used this combination to show that BPA and E2 commonly regulate major metabolic pathways, but results suggest the existence of different mechanisms for BPA and E2 action. This methodology should be useful to better understand mode of actions and define adverse outcome pathways (OAP).

In their original research, Khalil et al. exposed CD1 mice during pregnancy (prenatal group) or during lactation (postnatal group) to the flame retardant 2,2′,4,4′-tetrabromodiphenylether (BDE-47) and studied different endpoints in 10-month-old male offspring. The authors find similar results in both groups after studying liver histology, transcriptome, and liver-blood triglyceride balance. Interestingly, they find opposite effects of low and intermediate doses in the expression of important metabolism-related genes such as CD36, that may explain the observed shifts in blood triglyceride levels.

## Reviews

In their review, Papalou et al. looked at the effects MDCs have during specific developmental windows on multiple systems involved in metabolism specifically from epigenetic changes induced by MDC exposures during development. An important aspect of these MDC-induced epigenetic changes is their capacity to affect the germ line, passing those changes to subsequent generations and leading to metabolic diseases such as obesity, diabetes, and NAFLD.

On the topic of obesity, Heindel reviewed the history of “obesogen,” those MDCs that promote the development of obesity. In his historical analysis of obesogenic MDCs, Heindel went back over 17 years ago when the first article linking environmental chemicals with obesity was published. Shortly after this founding paper, another research group proposed that obesity could be due in part to MDCs exposure. He then goes on to discuss subsequent work implicating MDC exposures during development on the later life onset of obesity.

Rubin et al. identify and discuss the factors underlying the diversity of phenotypes obtained after animal treatment with BPA. In addition to factors like species, strain, sex, route of exposure, non-monotonic dose-response, and other variables, they also include theoretical reasons that are intrinsic to the experiment because each animal is a unique individual. They discuss evidence strongly suggesting that metabolic pathways are an early target of BPA and not a consequence of obesity. This evidence points to a consistent phenotype of altered glucose homeostasis yet a more inconsistent obesity phenotype. Nonetheless, an important conclusion is that phenotypes are reproduced when conditions are equivalent.

Taking a lifespan approach, Tudurí et al. review the extensive data linking the ubiquitous MDC BPA on diabetes risk. The authors specifically examine the mechanisms linking BPA with metabolic dysfunction as well as how exposures during different life stages disrupt metabolism. Importantly, these data make clear that BPA exposure during sensitive windows of development induces metabolic dysfunction, including *in utero* and early postnatal life, adulthood, and pregnancy. Given the widespread exposure to BPA across populations, it is increasingly clear that efforts are required limit contact with this MDC across the lifespan.

Priyam et al. open an interesting new area of endocrine disruption in their discussion of the hormone-disrupting properties of engineered nanomaterials, including evidence linking them with disruptions in hormonal pathways and metabolic tissues that may lead to increased diabetes risk.

Numerous reports have linked type 2 diabetes mellitus (T2DM) to MDC exposures. This link was reviewed by Carmean and Seino who focused on evidence that these toxicants can impair the ability of pancreatic β-cells failure to meet the insulin needs of an individual. Their analysis pointed out that in several regions of the globe, exposure to the MDC inorganic arsenic (iAs) is highly correlated with T2DM onset, and they explore the mechanisms by which iAs promotes β-cell dysfunction.

Hamanaka and Mutlu similarly expand concepts of MDCs by reviewing the links between particulate matter air pollution and cardiovascular disease. Examining both the mechanistic data and epidemiological data, the authors illuminate the role air pollution plays in promoting metabolic and vascular dysfunction that likely exacerbates metabolic disease development and its consequences.

In addition to our expanding notion of what toxicants pose metabolic disease risk, Kassotis and Stapleton delve into the underappreciated mechanisms by which MDCs promote metabolic dysfunction. Using chemicals used in nonconventional oil and gas extraction as well as household dust as examples, the authors also explore the metabolic importance of chemical mixtures that define the realities of human exposure. Finally, they discuss the potential for high-throughput screening tools (e.g. ToxCast) to inform our understanding of toxicant-induced metabolic disease risk.

Examining over fifteen years of cellular and animal studies as well as epidemiological work in humans, Sargis et al. note that the role that MDCs play in the metabolic disorders is finally being recognized. Now that the problem has been identified, how can the medical community intervene to reduce disease outcomes? This is addressed in the review by, Sargis et al. who give light to the clinical translation of MDC science to reduce impact of MDCs on the epidemics of diabetes mellitus, NAFLD, and obesity.

## Mini-Reviews

Chamorro-Garcia and Blumberg in this mini-review discuss the current experimental approaches to mechanistically study how the prototypical obesogen, tributyltin (TBT), functions. They review the advantages and limitations of tools used from *in vitro* cellular approaches to transgenerational *in vivo* approaches, including epigenetics. The authors include the description of a new mechanism for the transgenerational transmission of epigenetic information.

Marraudino et al., review the effects at hypothalamic level of three different MDCs: Genistein, BPA and TBT. They describe the alterations that each of these chemicals produce on the neuronal circuits controlling food intake and energy balance. The effects are complex and they can be either orexigenic or anorexigenic depending on the MDC, sex and dose. This mini-review helps to clarify this highly important yet greatly unknown area of research.

Le Magueresse-Battistoni et al., this minireview summarizes evidence of the so-called cocktail effect on metabolic disturbances. It is described how low levels of multiple chemicals trigger metabolic alterations when the same concentration of these individual chemicals produced no effect. The authors discuss the need of including mixture effects into risk assessment.

In an important mini-review, Howard explores the emerging evidence linking MDCs with type 1 diabetes (T1D), including toxicant effects on pancreatic β-cells and immune function. Given the persistently cryptic originals of T1D and its rising prevalence, this manuscript raises important questions about the environmental origins of the disease.

Specifically examining the organochlorine pesticide dichlorodiphenyltrichloroethane (DDT) and its metabolite dichlorodiphenyldichloroethylene) DDE, Elmore and La Merrill review the evidence that these MDCs disrupt mitochondrial function. This mitotoxicity extends our understanding of the potential mechanisms by which toxicant exposures can promote metabolic dysfunction.

Taken together, this compilation of work demonstrates the diverse molecular and physiological consequences of MDC exposures while illuminating the potential role that exposures to these chemicals may have in the pathogenesis of multiple metabolic diseases, including obesity, diabetes, NAFLD, dyslipidemia, and cardiovascular disease. Importantly, this special issue highlights the diverse array of data linking MDC exposures with these complex disorders. Of critical importance, this special issue highlights the need of further research directed at understanding the modes-of-action of MDCs on their targets, the consequences of exposure on disease development, and the best practices for intervention to counter the adverse metabolic effects of MDC exposures. Given that the number of MDCs continues to rise and we are just now starting to appreciate and understand their metabolic toxicity, it is hoped that this Special Issue will spur future reports that clarify the links between MDC exposure and metabolic disease risk while defining a path forward for reducing the impact of this modifiable risk factor on the pandemic of metabolic disease plaguing the world.

## Author Contributions

All authors listed have made a substantial, direct and intellectual contribution to the work, and approved it for publication.

### Conflict of Interest

RS has received honoraria from CVS/Health and American Medical Forum. The remaining authors declare that the research was conducted in the absence of any commercial or financial relationships that could be construed as a potential conflict of interest.
